# A Framework for Mining Actionable Navigation Patterns from In-Store RFID Datasets via Indoor Mapping

**DOI:** 10.3390/s150305344

**Published:** 2015-03-05

**Authors:** Bin Shen, Qiuhua Zheng, Xingsen Li, Libo Xu

**Affiliations:** 1 Ningbo Institute of Technology, Zhejiang University, Ningbo 315100, China; E-Mails: tsingbin@nit.zju.edu.cn (B.S.); lixs@nit.zju.edu.cn (X.L.); 2 School of Computer Science and Technology, Hangzhou Dianzi University, Hangzhou 310018, China; E-Mail: zheng_qiuhua@163.com (Q.Z.)

**Keywords:** RFID, indoor mapping, shopping transaction path mining, data preprocessing, filtering redundant patterns, framework

## Abstract

With the quick development of RFID technology and the decreasing prices of RFID devices, RFID is becoming widely used in various intelligent services. Especially in the retail application domain, RFID is increasingly adopted to capture the shopping tracks and behavior of in-store customers. To further enhance the potential of this promising application, in this paper, we propose a unified framework for RFID-based path analytics, which uses both in-store shopping paths and RFID-based purchasing data to mine actionable navigation patterns. Four modules of this framework are discussed, which are: (1) mapping from the physical space to the cyber space, (2) data preprocessing, (3) pattern mining and (4) knowledge understanding and utilization. In the data preprocessing module, the critical problem of how to capture the mainstream shopping path sequences while wiping out unnecessary redundant and repeated details is addressed in detail. To solve this problem, two types of redundant patterns, *i.e.*, loop repeat pattern and palindrome-contained pattern are recognized and the corresponding processing algorithms are proposed. The experimental results show that the redundant pattern filtering functions are effective and scalable. Overall, this work builds a bridge between indoor positioning and advanced data mining technologies, and provides a feasible way to study customers’ shopping behaviors via multi-source RFID data.

## 1. Introduction

Nowadays, various types of sensors, such as Kinect sensor [1], inertial motion units [2], ultrasound range sensor [2], GPS [3], Radio Frequency Identification (RFID), laser scanners [4] and remote sensor networks [5], have been used to perceive the physical environment, and many promising solutions [2,6,7] have been proposed to realize the effective mapping from the physical world to the cyberspace. Among them, RFID technology is one of the most promising technologies. Based on geometric mapping, it has been increasingly used in various application scenarios, such as intelligent exhibition halls, goods tracking and congestion detection in logistics and distribution [8], abnormal activity detection and insecurity factor detection in access control, and so on.

In the grocery industry, when various items, not only returnable transport carts, trolleys and kegs, but also valuable products, are equipped with RFID tags, item-level RFID infrastructures are established [9]. They can be utilized to realize a wide range of smart applications, e.g., auto check-outs [9], item-level valuable merchandise tracking [10], vendor managed inventory [11], smart price tags [9], *etc.* [12,13]. Among them, one of the more attractive applications is tracking customers’ shopping paths. These paths can be captured based on identifying the moving trajectories of smart shopping carts/trolleys/keys which are tagged with RFID [14]. Besides that, shopping carts/trolleys/keys featuring RFID readers can recognize valuable products put into the carts/trolleys/keys, if each valuable product is tagged with an RFID label. As a result, both the walking trajectories of customers and the corresponding purchase behaviors are automatically recorded in the RFID datasets, which are quite precious for mining in-depth knowledge about the shopping behaviors of customers.

Clearly, in the competitive retail climate, discovering insights from the shopping behaviors of customers and then turning these insights into promotion and customer care actions are crucial for enhancing retail business and service quality [15]. Towards this vision, one fundamental work is to study customers’ shopping paths in conjunction with their purchasing behaviors. The quick development of in-door positioning [6,7,16,17,18,19,20,21,22,23] and data mining [24,25,26,27,28,29,30,31,32] technologies sheds light on the above problem, and motivates us to consider building a bridge between RFID-based indoor mapping and advanced data mining techniques to explore customer’s shopping behavior in depth.

Consequently, in this study, we propose a framework for mining actionable navigation patterns using multi-source RFID data, *i.e.*, shopping path data and RFID-supported customers’ purchasing behavior data. Actionable navigation pattern [24,25] is very useful for understanding behaviors of customers and can be applied to various applications, such as customer navigation, active advertising and recommendations, *etc.* In this framework, we first use the path graph to map the problem in the physical space to a problem in the cyber space, where shopping paths are represented by sequences of path segments. After the mapping between the physical space and the cyber space, the problem of RFID-based shopping path analytics is converted to sequential pattern analysis [26], which has plenty of research in data mining field [27,28,29,30,31,32] for further reference.

This paper is organized as follows: first, we introduce indoor mapping technologies and related terms in Section 2 and Section 3, respectively. Then, in Section 4, the framework for mining multi-source in-door RFID data is presented, and four modules are discussed in detail, which are: (1) mapping from the physical space to the cyber space; (2) data preprocessing; (3) pattern mining and (4) knowledge understanding and utilization. In Section 5, we address a key problem existing in the data preprocessing module, which is how to identify the mainstream shopping transaction paths while wiping out unnecessary redundant and repeated details. An algorithm which can filter two types of redundant patterns is also proposed. Then, a simulated shopping path generator is discussed in Section 6, and the experimental evaluation of the algorithm is given in Section 7. Finally, we discuss the contributions towards a real supermarket scenario and conclude our work in Section 8 and Section 9, respectively.

## 2. Indoor Mapping

To easily comprehend our proposed framework, we provide below a broad overview of indoor mapping technologies.

### 2.1. Overview

With the progress in sensor technology, many promising indoor-mapping solutions [1,2,3,6,7,16,17], which can provide precise (or proximity), reliable and robust positioning services, have been proposed. Commonly, an indoor-mapping solution contains two components: (1) a physical-layer for sensing and (2) a software-realized data processing and location positioning, where the sensing capability is based on various available technologies, such as ultra-wideband (UWB), RFID, wireless local area network (WLAN), Bluetooth, ultrasound and video cameras, or the combination of these technologies [6,16,17]. On the basis of sensing, location positioning can be achieved using positioning algorithms, which can be mainly divided into three categories: triangulation, scene analysis and proximity [17]. Triangulation schemes employ geometrical property-based techniques, which are typically time of arrival (TOA), time difference of arrival (TDOA), round-trip time-of-flight (RTOF), angle of arrival (AOA) and received signal strength (RSS) [6]. Scene analysis approaches commonly involve two phases: an offline phase of training and an online phase of positioning. In the offline phase, fingerprints of scenes are collected and stored; during the online recognition phase, machine learning methods (e.g., extreme learning machine [18]) are adopted to compare the observed fingerprints with pre-measured fingerprints for position determination [17].

### 2.2. RFID-Based Indoor Positioning

Among the above technologies, RFID is an attractive option for coarse grained localization which provides proximity position information, because it is relatively cost-effective and is quite suitable for tracking a large number of items. Therefore, RFID technology is selected in our application of tracking shopping carts and purchased items in a supermarket.

Non-contact RFID positioning systems include three components: RFID readers, tags and servers, where tags can be active or passive. Active RFID tags equipped with internal batteries can broadcast their signals initiatively, and provide a much longer signal transmission range than passive tags; while passive tags are powered by signals transmitted from RFID readers [19,22]. Several basic frequency bands are employed by RFID systems, which include low frequency (LF), high frequency (HF), very high frequency (VHF), ultra-high frequency (UHF) and microwave frequency. Different frequency bands offer different read ranges which normally vary from 10 cm to 12 m, and are suited for different applications [20]. Representative RFID-based precise location sensing systems are SpotON [21] and LANDMARC [22], where reference tags are employed as landmarks. Typical work towards RFID- based proximity positioning includes tracking materials on construction job sites by combining proximity reads from a discrete range [23]. 

Fault tolerance is another important issue for RFID-based positioning system. The faults (false positive/negative readings) may be caused by many factors, such as hardware failures (e.g., malfunction, running out of battery energy), multipath interference, or complex radio propagation [33]. Countermeasures can be divided into two categories: physical solutions that are based on hardware performance improvement [34], and intelligent software solutions that are based on spatial-temporal correlations/redundancy [33,35].

## 3. Materials for the Study

In this section, related concepts are defined, and the notations used in this study are summarized in Table 1.

*Definition 1.* A *path segment s* is a directed edge associated with a direction symbol (*s.dir*), two terminal points (one is the start terminal point *s.b* and the other is the end terminal point *s.e*), and its length (*s.l*). The path segment only can be travelled from *s.b* to *s.e*. The reverse-order path segment of *s* is the path segment sharing the same edge with *s* but reverse direction, *i.e.*, *s_reverse_*, where *s_reverse_.dir* and *s.dir* are reverse, *s_reverse_.b* equals *s.e*, *s_reverse_.e* equals *s.b*, and *s_reverse_.l* and *s.l* are equal.

*Definition 2.* A *path graph G* is a directed graph, *i.e.*, *G* = (*V*, *E*), where *V* is the set of terminal points of path segments, and *E* is the set of path segments. Path graph *G* is an abstraction of the connections of path segments in a real field.

*Definition 3.* A *shopping path SP* is a sequence of path segments, *SP* = <*s*_1_, *s*_2_,…, *s_n_*>, where *s*_i+1_.*b* = *s_i_*.*e* and *s_j_* ∈ *E*, (1 ≤ *i* ≤ *n*−1, 1 ≤ *j* ≤ *n*). The beginning point and the ending point of *SP* can be represented as *SP*.*b* = *s*_1_.*b* and *SP*.*e* = *s_n_*.*e*.

*Definition 4*. There are several concepts related to shopping paths as given below:

(1) Given two shopping paths, *i.e.*, *SP* = <*s*_1_, *s*_2_,…, *s_n_*> and *SP'* = $<s_{1}^{\prime },s_{2}^{\prime }\ldots s_{l}^{\prime }>$ (*l*≤*n*), if there exists *i*, such that $s_{1}^{\prime }$ = *S_i_*, $s_{2}^{\prime }$ = *s*_*i*+1_, …, $s_{l}^{\prime }$ = *s*_*i*+*l*−1_, then *SP* is a super-sequence of *SP'*, and *SP'* is a subsequence of *SP* (denoted as *SP'* ⊂ *SP*). We also call that *SP'* is contained in *SP*.

(2) A navigation pattern *NP* means a subsequence of a shopping path.

(3) For a shopping path *SP* = <*s*_1_, *s*_2_,…,*s*_*k*+1_,…,*s_n_*>, the reverse-order path of *SP* is *SP_reverse_* =<*s_n,reverse_*,…,*s*_*k*+1,reverse_, *s*_*k*,reverse_,*s*_2*reverse*_, *s*_1*reverse*_>, where *s_i,reverse_* (1≤*i*≤*n*) is the reverse-order path segment of *s_i_*.

(4) Given a shopping path *SP* = <*s*_1_, *s*_2_,…,*s_k_*, *s*_*k*+1_,…,*s_n_*>, *SP_prefix_* = <*s*_1_, *s*_2_,…,*s_k_*>, is called a **prefix** of *SP*, and *SP_prefix_* = <*s*_*k*+1_, *s*_2_,…, *s_n_*> is called a suffix of *SP*, where 1≤*k*≤*n*. 

(5) Given *n* shopping paths, *i.e.*, *SP*_1_, *SP*_2_, …, *SP*_*n*-1_ and *SP_n_*, if *SP_i_*.*e* = *SP*_*i*+1_.*b* (1 ≤ *i* ≤ *n*−1) is satisfied, these shopping paths can be connected one after another, and the connection can be marked as *SP*_1_→*SP*_2_→…→*SP_n_*.

**Table 1:** Notations.

Notation	Description
*s, s_i_*	A path segment
*s_reverse_*	The reverse-order path segment of *s*
*s_i_.b, s_i_.e*	The start terminal point, the end terminal point of *s_i_* respectively
*t_i_*	Unit time per unit length spent in *s_i_*
*v_i_*	A terminal point
*T_i_*	The itemset purchased in *s_i_*
*G*	A path graph
*SP, SP', SQ*	A shopping path
*SP'* ⊂ *SP*	SP is a super-sequence of *SP'*, and *SP'* is a subsequence of SP
*STP, STP_prefix_, STP_suffix_*	A shopping transaction path
Trans(*STP*)	Transforming *STP* to a shopping path
*i_item_*	An item
*i_item_* < *STP*	*i_item_* is purchased in *STP*
*D*	A shopping transaction path database
*S_item_*, |*S_item_*|	A set of items, and the number of its elements respectively
*Γ_s_*	The itemset sold in s
*SIT*	The Segment-Item Table
*E_item_*	The set of path segments that sell *i_item_*
*IST, LT, PT*	The Item-segment table, the Length table, and the Path-set table respectively

*Definition 5.* Given a shopping path *SP*, the connection between *SP* and its reverse-order path *SP_reverse_* (*i.e.*, *SP*→*SP_reverse_*) forms a symmetric pattern. If *SP*.*b* = *SP*.*e*, *SP* is called a loop pattern. Given a loop pattern *SP*, if *SP* repeats *n* (*n* ≥ 2) times successively, *i.e.*, *SP*→*SP*→…→*SP*, we call it a loop repeat pattern. Given a shopping paths, *i.e.*, *SP*, we call the pattern *SP*→*SP_reverse_*→*SP* a *palindrome-contained pattern*.

*Definition 6.* A *shopping transaction path* is a sequence of triples, *STP* = <(*s*_1_,*t*_1_,*T*_1_), (*s*_2_,*t*_2_,*T*_2_), …, (*s_n_*,*t_n_*,*T_n_*)>, where (*s_i_*, *t_i_*, *T_i_*) means that a shopper purchases the itemset *T_i_* and spends *t_i_* unit time per unit length in the path segment *s_i_* (1≤*i*≤*n*).

*Definition 7.* Given a shopping transaction path, *i.e.*, *STP* = <(*s*_1_,*t*_1_,*T*_1_), (*s*_2_,*t*_2_,*T*_2_), …, (*s_n_*,*t_n_*,*T_n_*)>, there are several concepts, which are relevant to shopping transaction paths, are given below:

(1) For simplicity, all *s_i_* (1≤*i*≤*n*) are called the path segments of shopping transaction path *STP*.

(2) For a given item *i_item_*, if *i_item_* ∈ *T*_1_∪*T*_2_∪…∪*T_n_*, we call *i_item_* is purchased in *STP*, *i.e.*, i_item_ < STP.

(3) Given a fragment of *STP*, *i.e.*, *STP'* = <(*s_k_*,*t_k_*,*T_k_*), (*s_k_*_+1_,*t_k_*_+1_,*T_k_*_+1_), …, (*s_l_*,*t_l_*,*T_l_*)>(1≤*k*≤*l*≤*n*), we called *STP'* a subsequence of *STP*, and *STP'* is contained in *STP*.

(4) *STP_prefix_* = <(*s*_1_,*t*_1_,*T*_1_), (*s*_2_,*t*_2_,*T*_2_), …, (*s_k_*,*t_k_*,*T_k_*)>, is called a prefix of *STP*, where 1<*k*<*n*. *STP_suffix_* = <(*s_k_*_+1_,*t_k_*_+1_,*T_k_*_+1_), (*s_k_*_+2_,*t_k_*_+2_,*T_k_*_+2_), …, (*s_n_*,*t_n_*,*T_n_*)> is called a suffix of *STP*.

(5) Given a shopping transaction path, *STP* = <(*s*_1_,*t*_1_,*T*_1_), (*s*_2_,*t*_2_,*T*_2_), …, (*s_n_*,*t_n_*,*T_n_*)>, if only path segments appear in *STP*, we transform it to a shopping path, *SP* = <*s*_1_, *s*_2_,…, *s_n_*>, which is called the shopping path of *STP* and denoted as *SP* = Trans(*STP*).

(6) Given *n* shopping transaction paths, *i.e.*, *STP*_1_, *STP*_2_, …, *STP_n_*_-1_ and *STP_n_*, if Trans(*STP_i_*).*e* = Trans(*STP_i+_*_1_).*b* (1≤*i*≤*n*−1) is satisfied, these shopping transaction paths can be connected one after another, and the connection can be marked as *STP*_1_→*STP*_2_→…→*STP_n_*.

For example, *STP* = <(*s*_1_,1, Ø),(*s*_2_,0.8,Ø),(*s*_3_,8,{*i*_1_,*i*_2_}),(*s*_4_,0.8,Ø),(*s*_5_,5,{*i*_3_})> denotes that when the shopper visits *s*_1_, *s*_2_,…, *s*_5_ consecutively, he/she spends 1, 0.8, …, 5 unit time per unit length in these path segments respectively. Meanwhile, the shopper purchases {*i*_1_,*i*_2_} in *s*_3_ and purchases {*i*_3_} in *s*_5_. *s*_1_, *s*_2_,…, *s*_5_ are all path segments of *STP*. Among them, *s*_1_ is the first path segment of *STP*, *s*_2_ is the second one, …, and *s*_5_ is the last one. Because *i*_1_∈Ø∪Ø∪{*i*_1_,*i*_2_}∪Ø∪{*i*_3_}, we have *i*_1_ < *STP*. *STP* can be transformed to a shopping path, that is to say Trans(*STP*) = <*s*_1_, *s*_2_,…, *s*_5_>.

*Definition 8.* A *mainstream of shopping path* is a shopping path without containing any loop repeat or palindrome-contained subsequence pattern. A shopping transaction path *STP* is a *mainstream of shopping transaction path*, if Trans(*STP*) is a mainstream of shopping path.

*Definition 9.* A *Segment-Item Table* (*SIT*), maintaining the information of items sold in each path segment, is denoted as below:

*SIT* = {(*s*_1_, *Γ*_*s*_1__), (*s*_2_, *Γ*_*s*_2__), …, (*s_W_*, *Γ_s_W__*)} (1)

where *s_i_* is a path segment, *Γ_s_i__* is the itemset sold in *s_i_* (1≤*i*≤*W*), and *W* is the total number of path segments.

*Definition 10.* An *Item-Segment Table* (*IST*), maintaining the information about the segments where each item is sold, is denoted as below:

*IST* = {(*i_item,1_*, *E_item_*_,1_), (*i_item,2_*, *E_item_*_,2_), …, (*i_item,U_*, *E_item,U_*)} (2)

where *i_item,j_* is an item, *E_item,j_* is the set of path segments that sell *i_item,j_* (1≤*j*≤*U*), and *U* is the total number of items.

*Definition 11.* A *Length Table* (*LT*), maintaining the length information of path segments, is denoted as below:

*LT* = {(*s*_1_, *s*_1_*.l*), (*s*_2_, *s*_2_*.l*), …, (*s_W_*, *s_W_.l*)} (3)

where *s_i_* is a path segment, and *s_i._.l* is the length of *s_i_* which can be obtained according to the length of normal trajectory of *s_i_* (1 ≤ *i* ≤ *W*).

## 4. System Framework of RFID Path Explorer

In this section, we describe the framework of the RFID supported paths and behaviors mining, called *RFID Path Explorer*.

**Figure 1:**
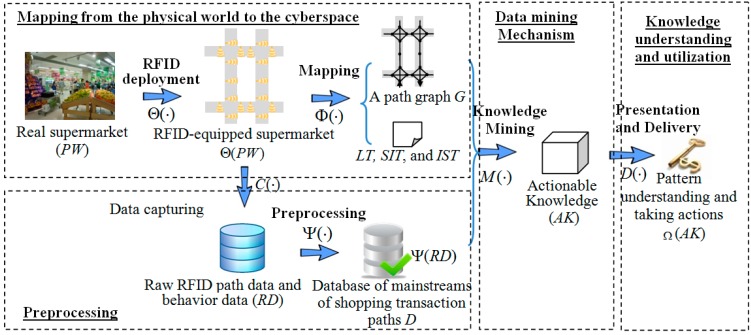
The framework for RFID based shopping transaction path mining.

The proposed framework consists of four modules: (1) mapping from the physical world to the cyber space; (2) data preprocessing; (3) a data mining mechanism; and (4) knowledge understanding and utilization (see Figure 1). Table 2 shows an example of shopping transaction path database which contains five shopping transaction paths. Below, we explain them in detail.

**Table 2:** An example of shopping transaction path database.

*STP_id_*	Shopping Transaction Path
1	(AB, 0.8, Ø), (BC, 1, Ø), (CD, 4, {i_1_, i_2_}), (DE, 3, {i_3_}), (EF, 0.8, Ø), (FD, 0.8, Ø), (DK, 0.8, Ø)
2	(AB, 0.9, Ø), (BC, 1, Ø), (CD, 5, {i_1_}), (DK, 0.8, Ø)
3	(DK, 0.9, Ø), (KC, 0.8, Ø), (CD, 5, {i_2_}), (DE, 5, {i_3_})
4	(BC, 0.8, Ø), (CD, 4, {i_1_}), (DK, 1, Ø), (KA, 0.8, Ø), (AD, 1, Ø), (DE, 4, {i_3_}), (EF, 1, Ø)
5	(DK, 0.9, Ø), (KC, 1, Ø), (CD, 6, {i_2_, i_4_}), (DK, 1, Ø)

### 4.1. Indoor Mapping from the Physical World to the Cyberspace

The module of indoor mapping from the physical world (*PW*) to the cyber space consists of two steps as shown in Figure 1.

#### 4.1.1. Step 1. RFID Deployment (Θ(·))

According to the task of application domain (*i.e.*, finding actionable navigation patterns for purchasing an item), suitable RFID devices should be chosen and deployed in a real field (*i.e.*, real supermarket). For instance, in our application, a RFID tag, which has a unique Electronic Product Code (*EPC*), is attached to each shopping trolley. RFID readers are located at various places of a supermarket, such as the entrance, the checkout, the gathering place for shopping carts, aisles and thoroughfares *etc.*, and used to identify shopping trolleys passing by. At the same time, when valuable items attached with RFID tags are put into a shopping trolley, they also can be recognized by this RFID-reader-equipped shopping trolley. Thus, both shoppers’ path and behaviors can be captured (*C*(·)) and recorded. For the sake of robust, redundant multiple readers/tags [23] and received signal strength functions of RFID devices [21] can be added to promote the reliability of proximity location determination.

#### 4.1.2. Step 2. Mapping (Φ(·))

**Figure 2:**
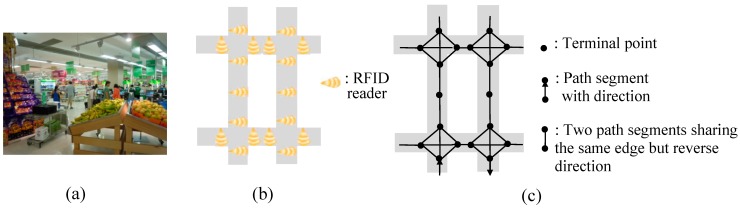
An illustration of path graph (**a**) A photo of Real supermarket. (**b**) An illustration of a part of supermarket after RFID deployment. (**c**) An illustration of a part of path graph after mapping.

Recorded raw RFID data can’t be understood without the support of semantic information about these data. Therefore, attributes and features related to the analysis task should be abstracted from the physical world and mapped to the cyber-space. In the context of our application, a path graph *G* is used to abstract the connections of path segments, after RFID devices are deployed in a supermarket. An illustration of path graph is shown in Figure 2c, which is mapping from Figure 2b. A segment-item table (*SIT*) and an item-segment table (*IST*) are also extracted to reflect the items sold in each segment and the segments where each item is sold respectively. An example of *SIT* and *IST* is shown in Table 3.

**Table 3:** An example of *SIT* and *IST*.

Path Segment	Itemset	Itemset	Path Segment
AB	NULL	*i*_1_	{CD, PZ}
BC	NULL	*i*_2_	{CD, DE}
CD	{*i*_1_, *i*_2_, *i*_6_*, i*_11_, *i*_12_}	*i*_3_	{GH, PQ, PZ}
DE	{*i*_2_, *i*_15_}	*i*_4_	{GJ}
…	…	…	…
PZ	{*i*_1_, *i*_3_, *i*_20_, *i*_5000_}	*i*_5000_	{PZ}

### 4.2. Preprocessing

**Figure 3:**
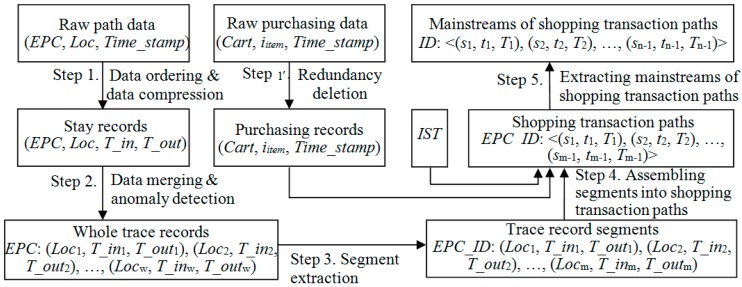
RFID data preprocessing.

After the raw RFID path and behavior data is captured, the preprocessing is shown in Figure 3.

#### 4.2.1. Step 1. Data Ordering and Data Compression

Raw RFID path data has the form (*EPC*, *Loc*, *Time_stamp*), where *EPC* is the Electronic Product Code of the tag that uniquely represent a shopping cart, *Loc* is the identification location whose reader finds the tag, and *Time_stamp* is the time when the RFID reading takes place [36]. These raw data firstly need to be sorted on *EPC* and *time*, and then be transformed to the form of stay record, *i.e.*, (*EPC*, *Loc*, *T_in*, *T_out*), where *T_in* is the time when the RFID tag enters the identification area, and *T_out* is the leave time [37].

When an item (*i.e.*, *i_item_*) is put into a shopping cart (*i.e.*, *Cart*), a raw purchasing data (*i.e.*, (*Cart*, *i_item_*, *Time_stamp*)) is also generated, where *Time_stamp* is the time of detecting the item. And then, raw purchasing data is continuously produced, until the item is picked out of the shopping cart. Therefore, for an item, only the record of first reading needs to be saved, which marks purchasing of the item happens.

#### 4.2.2. Step 2. Data Merging and Anomaly Detection

For a *EPC*, from the stay records, a whole trace record can be constructed, which has the form *EPC*: (*Loc*_1_, *T_in*_1_, *T_out*_1_), (*Loc*_2_, *T_in*_2_, *T_out*_2_), …, (*Loc_w_*, *T_in_w_*, *T_out_w_*), where *Loc_i_* is the location where the tag is detected, *T_in_i_* and *T_out_i_* are the entering time and the leaving time of *Loc_i_* respectively, and (*Loc_i_*, *T_in_i_*, *T_out_i_*) is ordered by *T_in_i_* (1 ≤ *i* ≤ *W*) [38]. For a whole trace record, if there are two same successive locations (*i.e.*, *Loc_i_* = *Loc_i+1_*), then (*Loc_i_*, *T_in_i_*, *T_out_i_*) and (*Loc_i+_*_1_, *T_in_i+_*_1_, *T_out_i+_*_1_) can be merged to (*Loc_i_*, *T_in_i_*, *T_out_i+_*_1_). Thus we have a whole trace record where any two successive locations are different.

Here, any two consecutive locations are required to meet spatial constraint [35] that these two locations should be directly connected in the path graph. Two successive locations which cannot satisfy this constraint are labelled as an anomaly, and the anomaly should be checked further to infer/determine whether missed readings or false positive readings occur. If permanent/intermittent/transient faults leading to this anomaly can be identified, missed readings are filled in and false positive readings are discarded to make the whole trace record smoothly connected; otherwise, suspicious readings are removed and the remaining parts of the trace record are kept separately. This prior-knowledge based validation mechanism can further promote the reliability of sensing.

#### 4.2.3. Step 3. Segment Extraction

In the supermark scenario, shopping carts are recycled and used by different shoppers at different shopping times. Thus, the whole trace record of a certain *EPC*, which is the proxy of a cart, commonly contains multiple shopping trips of various shoppers. Besides, path sequences of supermarket staff collecting shopping carts may also be contained. Therefore, in order to study customers’ shopping behavior, it is necessary to extract individual shopping trips from the whole trace records.

**Figure 4:**
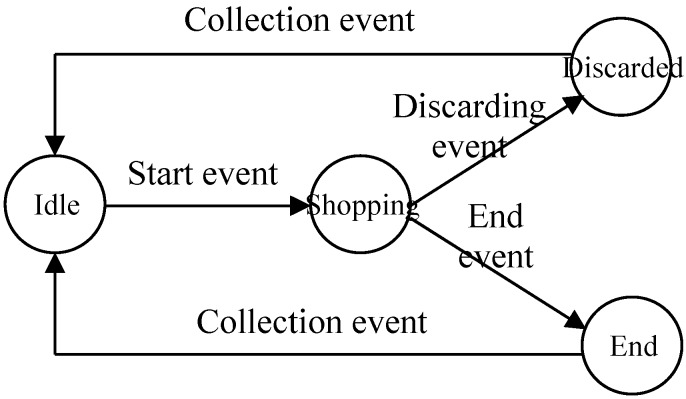
The finite state machine model for shopping carts.

For the above purpose, we develop a finite state machine model [39] for shopping carts (see Figure 4) by referring to the real situation in supermarkets. In Figure 4, there are four states for a shopping cart: “idle”, “shopping”, “discarded” and “end” states. The “idle” state implies that the cart stays in the gathering place for shopping carts and is currently available. The “shopping” state indicates that the cart is in use by a shopper. The “discarded” state means that the cart is discarded midway by a shopper, and the “end” state implies the cart arriving at the checkout and the end of a shopping trip.

In the model, a state will be transformed to another one, if a certain event [40] is triggered. The initial state of a cart may be “idle”. If a “start” event happens, the “idle” state will become the “shopping” state, where the “start” event can be defined as the observation that a shopping cart leaving the gathering place and entering the main entrance. Then, the “shopping” state will be changed to the state of “discarded” if the “discarding” event happens or to the “end” state if the “end” event takes place. Both the “discarded” and the “end” states are followed by the “collection” event, and will be transformed to the “idle” state again. Here, the “discarding”, the “end” and the “collection” events also should be defined according to the real situation. For example, we may define the “discarding” and the followed “collection” events as a long stay in a certain location and then moving to the gathering place directly, and the “end” event as a cart arriving checkout with items taken out of the shopping cart. Thus trace record segments can be extracted from a whole trace record, where each segment represents a single shopping trip.

#### 4.2.4. Step 4. Assembling Segments into Shopping Transaction Paths

In order to analyze shopping paths, terminal-points focused trace record segments need to be further transformed to path-segments focused shopping transaction paths, which also combine the information of purchased items. The transform process is shown below.

First, given a trace record segment of a shopping cart (*Cart*), *i.e.*, *EPC_ID*: (*Loc*_1_, *T_in*_1_, *T_out*_1_), (*Loc*_2_, *T_in*_2_, *T_out*_2_), …, (*Loc*_m_, *T_in*_m_, *T_out*_m_), and a length table (*LT*), the trace record segment can be converted to a shopping path with time information in the form of *EPC_ID*: (*s*_1_, *t*_1_), (*s*_2_, *t*_2_), …, (*s_m_*_-1_, *t_m_*_-1_), where *s_i_* is the path segment connecting two successive locations (*i.e.*, *Loc_i_* and *Loc_i+1_*), and *t_i_* represents the time spent in *s_i_* per unit length of *s_i_* (1 ≤*i* ≤ *m*−1). That is to say, *t_i_* equals *time_i_* / *s_i_,.l*, where *time_i_* representing the time spent in *s_i_* is assumed as (*T_in_i_*_+1_+*T_out_i_*_+1_)/2-(*T_in_i_*+*T_out_i_*)/2 for simplicity (1 ≤ *i*≤ *m*−1), and *s_i_*.*l* is the length of *s_i_*. Second, suppose the corresponding set of purchasing records of *Cart* is {(*Cart*, *i_item,1_*, *Time_stamp_item,1_*), …, (*Cart*, *i_item,n_*, *Time_stamp_item,n_*)}, where *T_in*_1_ ≤ *Time_stamp_item,j_* ≤ *T_out*_m_ (1≤*j*≤*n*). Then for each (*Cart*, *i_item,j_*, *Time_stamp_item,j_*), given *IST* = {(*i_item,1_*, *E_item,1_*),…,(*i_item,U_*, *E_item,U_*)}, there are four cases to decide which path segment *i_item,j_* is purchased in.

Case 1. If ∃ 2 ≤ *i* ≤ *m*-1, *T_out_i_* ≤ *Time_stamp_item,j_* ≤ *T_in_i+1_*, then obviously *i_item,j_* is purchased in *s_i_*.

Case 2. If *T_in_1_* ≤ *Time_stamp_item,j_* < *T_out_1_*, then obviously *i_item,j_* is purchased in *s_1_*; if *T_in_m_*<*Time_stamp_item,j_* ≤ *T_out_m_*, clearly, *i_item,j_* is purchased in *s_m-1_*.

Case 3. If ∃2 ≤ *i* ≤ *m*-1, *T_in_i_* < *Time_stamp_item,j_* < *T_out_i_*, *s_i_* ∈ *E_item,j_* and *s_i-_*_1_∉*E_item,j_*, then it can be deduced that *i_item,j_* is purchased in *s_i_*. If ∃2≤*i*≤*m*-1, *T_in_i_*<*Time_stamp_item,j_* < *T_out_i_*, *s_i_* ∉ *E_item,j_* and *s_i_*_-1_∈*E_item,j_*, then it can be derived that *i_item,j_* is purchased in *s_i_*_-1_.

Case 4. If ∃2 ≤ *i* ≤ *m*-1, *T_in_i_* < *Time_stamp_item,j_* < *T_out_i_*, *s_i_* ∈ *E_item,j_* and *s_i-_*_1*∈*_*E_item,j_*, we can’t judge which path segment *i_item,j_* is purchased in among *s_i-_*_1_ and *s_i_*. In this case, besides the above conditions are met, if *T_in_i_* < *Time_stamp_item,j_*≤(*T_in_i_+T_out_i_*)/2, *i_item,j_* is most probably purchased in *s_i-_*_1_; otherwise, if (*T_in_i_+T_out_i_*)/2 < *Time_stamp_item,j_* < *T_out*, *i_item,j_* is most likely purchased in *s_i_*.

Thus, after all items (*i.e.*, *i_item,j_* (1 ≤ *j* ≤ *n*)) are added to the corresponding item set of path segment where this item is purchased, we can obtain a shopping transaction path, *i.e.*, *EPC_ID*: <(*s*_1_, *t*_1_, *T*_1_), (*s*_2_, *t*_2_, *T*_2_), …, (*s*_m-1_, *t*_m-1_, *T*_m-1_)>, where *T_i_* (1 ≤ *I* ≤ *m*-1) is the item set purchased in *s_i_*.

#### 4.2.5. Step 5. Extracting Mainstreams of Shopping Transaction Paths

In the context of web browsing, Chen *et al.* [27] first introduced the concept of maximal forward reference, and proposed a method of breaking a user session down into several maximal forward references if backward references appear in this session. However, several limitations still exist in the scheme of extracting maximal forward reference.

First, their extraction method assumes that backward references are all for easy of travelling, and not for browsing. But this assumption fails in the context of a real supermarket. There are two intentions for a shopper choosing to go backward. One is trying to explore and purchase in the backward reference, and the other is going through the backward reference to other interested sections, so the method of Chen *et al.* [27] can’t be applied here.

Second, their method throws away all backward references, which might provide important clues on shoppers’ purchasing and navigation behaviors.

**Figure 5:**
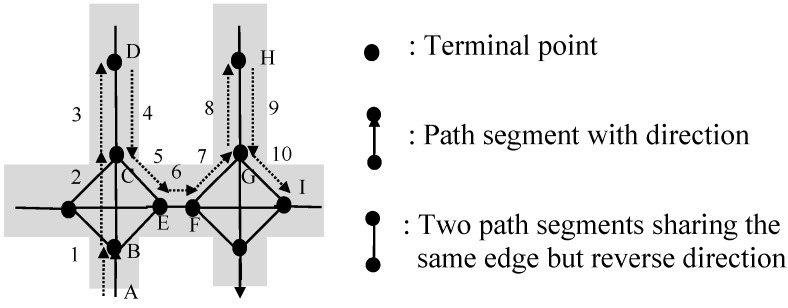
An example for identifying maximal forward reference.

Third, after applying their method, the frequency of prefix sequence in front of symmetric pattern is increased unexpectedly. For instance, suppose there is a shopping path < AB, BC, CD, DC, CE, EF, FG, GH, HG, GI > shown in Figure 5, where two symmetric patterns exist, *i.e.*, < CD, DC > and < GH, HG >. After applying the method of Chen *et al.* [27], the set of maximal forward shopping path is < AB, BC, CD >, < AB, BC, CE, EF, FG, GH >, < AB, BC, CE, EF, FG, GI >. We can find that the frequency of prefix sequence < AB, BC > unexpectedly becomes 3, while it is 1 in the original shopping path. The frequency of prefix sequence < AB, BC, CE, EF, FG > is converted to 2, while it is 1 in the original shopping path.

Fourth, a maximal forward reference terminates if a backward reference appears. Thus, a maximal forward reference will not contain any symmetric pattern. This may lead to an unexpected loss of important knowledge on symmetric pattern. For example, from the shopping path < AB, BC, CD, DC, CE, EF, FG, GH, HG, GI > shown in Figure 5, we know that this customer would like to go to the aisle of CD first, turn back to the main thoroughfare of EF, go forward to the aisle of GH, and then be back to the main thoroughfare again. The knowledge on customer’s turning back disappears in the set of maximal forward shopping path.

Therefore, instead of finding maximal forward references, we present another new scheme called *extracting mainstream of shopping transaction path*, which reserves necessary symmetric patterns, but discards redundant and repeated details. This scheme is discussed in the next subsection in detail.

### 4.3. Mainstreams of Shopping Transaction Paths

In the environment of a real supermarket, in order to choose items of interest, shoppers are inclined to push/pull shopping carts forward and backward. Symmetric patterns, loop patterns and redundant details may appear in shopping paths. In order to catch the mainstream of path sequences while discarding unnecessary redundant and repeated details, we put forward a scheme for identifying mainstream shopping transaction paths. In this scheme, we recognize that two types of redundant patterns need to be simplified, *i.e.*, loop repeat patterns and palindrome-contained patterns. .

#### 4.3.1. Processing of Loop Repeat Patterns

Successive repeated path sequence loops actually reflect the same shopping interest and share the same behavior pattern, so these loops can be combined into one loop. For a shopping path, we can compress several repeat loops into one directly. For a shopping transaction path with a loop repeat pattern, *i.e.*, *STP_prefix_*→*STP*_1_→*STP*_2_→…→*STP_n_*→*STP_suffix_*, where *STP_i_* (*i* = 1,…,*n*) shares the same navigation pattern, these *STP_i_* (*i* = 1,…,*n*) also can be combined into a single *STP_combine_* = <(*s*_1_, *t*_1_, *T*_1_), (*s*_2_, *t*_2_, *T*_2_),…, (*s_m_*, *t_m_*, *T_m_*)>, so we also need to consider specifying values of *t_j_* and *T_j_* in *STP_combine_* (*j* = 1,…,*m*). The time spent in a path segment is normally comprised of two parts: walking time and time for exploring and purchasing. We consider that if a shopper tries to complete the task of exploring and purchasing in one loop (say *STP_combine_*), which is previously done in multiple loops (say *STP_i_* (*i* = 1,…,*n*)), time spent in the same path segment of loop should be cumulated, and itemsets purchased in the same path segment but in different loops also should be combined.

Therefore, we have the following definition for simplification of loop repeat pattern:

*Definition 12.* A shopping path containing loop repeat pattern, *i.e.*, *SP_prefix_*→*SP*$\overset{n}{\to }$*SP*→*SP_suffix_*, can be simplified as *SP_prefix_*→*SP*→ *SP_suffix_*. A shopping transaction path containing loop repeat pattern, *i.e.*, *STP_prefix_*→*STP*_1_→*STP*_2_→…→*STP_n_*→ *STP_suffix_*, where *STP_i_* = <(*s*_1_, *t*_1,*i*_, *T*_1,*i*_), (*s*_2_, *t*_2,*i*_, *T*_2,*i*_),…, (*s_m_*, *t_m_*_,*i*_, *T_m_*_,*i*_)>, all *STP_i_* share the same navigation pattern (say Trans(*TSP*) = <*s*_1_, *s*_2_, …, *s_m_*>), and *m* is the number of path segments in *STP_i_* (*i* = 1,…,*n*), can be simplified as *STP_prefix_*→*STP_combine_*→ *STP_suffix_*, where *STP_combine_* = <(*s*_1_, *t*_1_, *T*_1_), (*s*_2_, *t*_2_, *T*_2_),…, (*s_m_*, *t_m_*, *T_m_*)>, *t*_walking_ is the smallest value of time spent per unit length in this shopping transaction path, *t_j_* and *T_j_* (*j* = 1,…,*m*) are defined as below:

(4) $$t_{j}=t_{\mathrm{walking}}+\sum_{i=1}^{n}t_{\mathrm{purchasing}}(j,i)\;(j=1,\ldots ,m)$$

*t*_purchasing_(*j*,*i*) = *t_j,i_* − *t*_walking_ (*i* = 1,…,*n*, *j* = 1,…,*m*) (5)

(6) $$T_{j}=\overset{m}{\underset{i=1}{\cup }}T_{j,i}\;(j=1,\ldots ,m)$$

**Figure 6:**
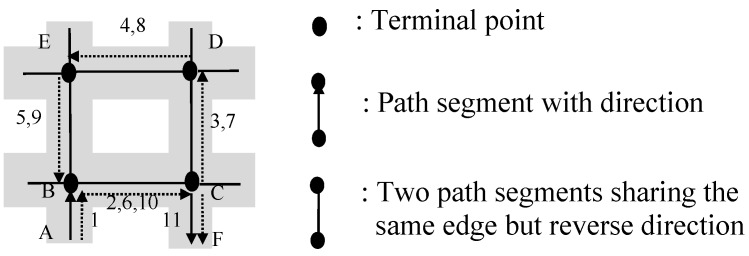
An illustrative example of loop repeat pattern.

For instance, for the shopping path <AB, BC, CD, DE, EB, BC, CD, DE, EB, BC, CF> shown in Figure 6, loop <BC, CD, DE, EB> appears two times continuously and forms a loop repeat pattern, so this shopping path can be simplified as a mainstream of shopping path <AB, BC, CD, DE, EB, BC, CF>.

For a shopping transaction path <(AB, 1, Ø), (BC, 2, Ø), (CD, 3, {*i*_1_}), (DE, 2, Ø), (EB, 6, {*i*_2_, *i*_3_}, (BC, 2, {*i*_4_}), (CD, 5, {*i*_6_, *i*_7_}), (DE, 1, Ø), (EB, 1, Ø), (BC, 1.1, Ø), (CF, 1, Ø)>, a loop repeat pattern <BC, CD, DE, EB>→<BC, CD, DE, EB> appears. Triple elements having the same path segment but different loops should be combined, *i.e.*, (BC, 2, Ø) and (BC, 2, {*i*_4_}), (CD, 3, {*i*_1_}) and (CD, 5, {*i*_6_, *i*_7_}), *etc.* We take the combination of (BC, 2, Ø) and (BC, 2, {*i*_4_}) as an example. First, we obtain that *t*_walking_ equals 1, which is the smallest one in the set of time spent per unit length in this shopping transaction path. Then, time spent in BC per unit length (*t*_BC_) is computed as 1 + (2−1) + (2−1) = 3, and itemset in BC (*T*_BC_) is Ø∪{*i*_4_} = {*i*_4_}. Therefore, the result of the combination of (BC, 2, Ø) and (BC, 2, {*i*_4_}) is (BC, 3, {*i*_4_}). Thus this shopping transaction path can be simplified as a mainstream of shopping transaction path < (AB, 1, Ø), (BC, 3, {*i*_4_}), (CD, 7, {*i*_1_, *i*_6_, *i*_7_}), (DE, 2, Ø), (EB, 6, {*i*_2_, *i*_3_}), (BC, 1.1, Ø), (CF, 1, Ø) >. This mainstream shopping transaction path retains the main movement of a shopper and prunes unnecessary details.

#### 4.3.2. Handling Palindrome-Contained Pattern

A palindrome-contained pattern, *i.e.*, *SP*→*SP_reverse_*→*SP*, is easily formed when a shopper compares the same kind of items in front of a shelf, and shows that shoppers hover in a *SP* navigation pattern. These three segments, *i.e.*, *SP*, *SP_reverse_* and the second *SP*, actually show the same shopping interest and share the same behavior pattern, so these successive segments can be simplified as *SP*. Thus, we have the following simplification method of palindrome-contained pattern 1:

*Definition 13*. A shopping path containing a palindrome-contained pattern, *i.e.*, *SP*→*SP_reverse_*→*SP*, can be simplified as *SP*. A shopping transaction path containing a palindrome-contained pattern, *i.e.*, *STP_prefix_*→*STP*_1_→*STP*_2_→*STP*_3_→*STP_suffix_*, where *STP_i_* = <(*s*_1_, *t*_1,*i*_, *T*_1,*i*_), (*s*_2_, *t*_2,*i*_, *T*_2,*i*_),…, (*s_m_*, *t_m_*_,*i*_, *T_m_*_,*i*_)> (*i* = 1, 3), *STP_2_* = <(*s_m_*_,*reverse*_, *t_m_*_,2_, *T_m_*_,2_), (*s_m_*_-1,*reverse*_, *t_m_*_-1,2_, *T_m_*_-1,2_), …, (*s*_1,*reverse*_, *t*_1,2_, *T*_1,2_)>, can be simplified as *STP_prefix_*→*STP_combine_*→*STP_suffix_*, where *STP_combine_* = <(*s*_1_, *t*_1_, *T*_1_), (*s*_2_, *t*_2_, *T*_2_),…, (*s_m_*, *t_m_*, *T_m_*)>, *t*_walking_ is the smallest value of time spent per unit length in this shopping transaction path, *t_j_* and *T_j_* (*j* = 1,…,*m*) are defined as below:

(7) $$t_{j}=t_{\mathrm{walking}}+\sum_{i=1}^{3}t_{\mathrm{purchasing}}(j,i)\;(j=1,\ldots ,m)$$

*t*_purchasing_(*j*,*i*) = *t_j,i_*− *t*_walking_ (*i*=1,2,3, *j* = 1,…,*m*) (8)

(9) $$T_{j}=\overset{3}{\underset{i=1}{\cup }}T_{j,i}\;(j=1,\ldots ,m)$$

**Figure 7:**
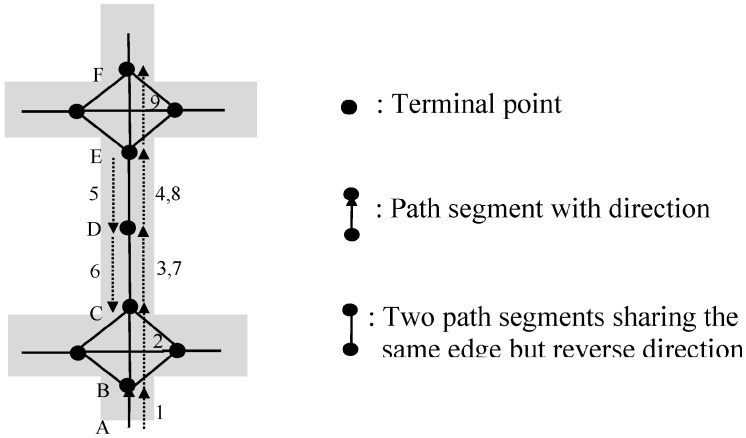
An illustrative example for palindrome-contained pattern.

For example, for the shopping path <AB, BC, CD, DE, ED, DC, CD, DE, EF> shown in Figure 7, there is a palindrome-contained pattern <CD, DE>→<ED, DC>→<CD, DE>, so this shopping path can be simplified as a mainstream of shopping path <AB, BC, CD, DE, EF>.

In a shopping transaction path <(AB, 1, Ø), (BC, 1, Ø), (CD, 3, Ø), (DE, 2, { *i*_1_}), (ED, 4, {*i*_2_, *i*_3_}), (DC, 6, { *i*_4_, *i*_5_, *i*_6_}), (CD, 5, {*i*_7_}), (DE, 1, Ø), (EF, 1, Ø)>, a palindrome-contained pattern <CD, DE>→<ED, DC>→<CD, DE> appears. Triple elements sharing the same edge (without considering the direction of path segments) in this palindrome-contained pattern can be combined, *i.e.*, (CD, 3, Ø), (DC, 6, { *i*_4_, *i*_5_, *i*_6_}) and (CD, 5, {*i*_7_}) can be combined into a single triple, and (DE, 2, { *i*_1_}), (ED, 4, {*i*_2_, *i*_3_}) and (DE, 1, Ø) also can be merged into one. Take the combination process of (CD, 3, Ø), (DC, 6, {*i*_4_, *i*_5_, *i*_6_}) and (CD, 5, {*i*_7_}) as an example. First, we know that *t*_walking_ is 1, which equals the smallest of time spent per unit length in this shopping transaction path. *t*_CD_ is computed as 1 + (3 − 1) + (6 − 1) + (5 − 1) = 12, and *T*_CD_ is Ø∪{*i*_4_, *i*_5_, *i*_6_}∪{*i*_7_} = {*i*_4_, *i*_5_, *i*_6_, *i*_7_}, so the result of the merging is (CD, 12, {*i*_4_, *i*_5_, *i*_6_, *i*_7_}). Thus, this shopping transaction path can be simplified as <(AB, 1, Ø), (BC, 1, Ø), (CD, 12, {*i*_4_, *i*_5_, *i*_6_, *i*_7_}), (DE, 5, {*i*_1_, *i*_2_, *i*_3_}), (EF, 1, Ø) >.

## 5. Algorithm for Identifying Mainstreams of Shopping Transaction Paths

The algorithm is an iterative process of filtering loop repeat patterns (*i.e.*, Function LRP_Filtering(STP)) and palindrome-contained patterns (*i.e.*, Function PCP_Filtering(STP)) for identifying mainstreams of shopping transaction path from shopping transaction paths, as shown in Algorithm 1.

**Algorithm 1** Identifying mainstreams of shopping transaction paths**Input**: Shopping transaction paths *D_STP_***Output**: Mainstreams of shopping transaction paths *D_MSTP_***Method**:For each shopping transaction path *STP* in *D_STP_* do { while (loop repeat patterns and palindrome-contained patterns exist in *STP*) do {Call LRP_Filtering(*STP*) to filter loop repeat patternsCall PCP_Filtering(*STP*) to filter palindrome-contained patterns } Add *STP* to *D_MSTP_* }Return *D_MSTP_*.

### 5.1. Function LRP_Filtering(STP)

Given a shopping transaction path *STP* = <(*s*_0_, *t*_0_, *T*_0_), (*s*_1_, *t*_1_, *T*_1_),…, (*s_n_*, *t_n_*, *T_n_*)>, the process of filtering loop repeat patterns is an iterative procedure to find the start position of the loop (say *µ*) and the number of path segments in the loop (say λ), such that *s_µ_*_+*i*_ = *s*_µ+λ+i_ (*i* = 0,1,…, λ − 1; µ+λ+*i* ≤ *n*). If *µ* and *λ* satisfying the above conditions are found, the fragments <(*s*_µ_, *t*_µ_, *T*_µ_),…, (*s*_µ+λ-1_, *t*_µ+λ-1_, *T*_µ+λ-1_)> and <(*s*_µ+λ_, *t*_µ+λ_, *T*_µ+λ_),…, (*s*_µ+2λ-1_, *t*_µ+2λ-1_, *T*_µ+2λ-1_)> in *STP* form a loop repeat pattern. This procedure is given in sub-function Find_RepeatLoops(*STP*), and three data structures (*i.e.*, path vector, hash table, and list of loop candidates) are used:

Definition 14. A *path vector* (say *PV*) is a vector of pair (*s*, *pos*), where *s* is a path segment and *pos* is the previous position of *s* in *PV*. A *hash table* (say *HT*) stores the current position (*i.e.*, *cur_pos_seg*) for each path segment (*i.e.*, *s*), and a hash function *f* is defined in *HT*, such that *HT*[*f*(*s*)] = *cur_pos_seg*. A *list of loop candidates* (say *List*) is a list of triple (*b_pos*, *e_pos*, *cur_pos*) representing a loop candidate (*i.e.*, the fragment <*PV*[*b_pos*].*s*, *PV*[*b_pos*+1].*s*, …, *PV*[*e_pos*].*s*>), where *cur_pos* is the current matching position between *b_pos* and *e_pos*. Based on these definitions, we have the following Function LRP_Filtering(*STP*), where the key part is the sub-function Find_RepeatLoops(*SP*).

**Function.** LRP_Filtering(*STP*)**Method:**While repeat loop pattern is found, do{(µ, λ, *n_loops*)←Find_RepeatLoops(trans(*STP*))If repeat loop pattern is found, do {For *STP*, combine *n_loops* fragments, *i.e.*, <(*s*_µ_, *t*_µ_, *T*_µ_),…, (*s*_µ+λ-1_, *t*_µ+λ-1_, *T*_µ+λ-1_)>, <(*s*_µ+λ_, *t*_µ+λ_, *T*_µ+λ_),…, (*s*_µ+2λ-1_, *t*_µ+2λ-1_, *T*_µ+2λ-1_)>, …, <(*s*_µ + (*n_loops*-1) × *λ*_, *t_µ_*_+(*n_loops*-1)×λ_, *T_µ_*_+(*n_loops*-1)×λ_),…, (*s_µ_*_+*n_loops*×λ-1_, *t_µ_*_+*n_loops*×*λ*-1_, *T_µ_*_+*n_loops*×*λ*-1_)>, to form a new *STP* according to Definition 12.} }Return *STP***Sub-Function.** Find_RepeatLoops(*SP*)Initialize *PV*, *HT* and *List* as emptySuppose *SP* = <*s*_0_, *s*_1_, …, *s_n_*>. For each path segment *s_i_* in *SP*, do {If *s_i_* is a key in *HT*, let *cur_pos_seg=HT*[*f*(*s_i_*)]; otherwise, insert a key-value pair (*s_i_*, “null”) to *HT* and let *cur_pos_seg*=“null”. Push the pair (*s_i_*, *cur_pos_seg*) onto *PV*, and get the position of this pair in *PV* (say *new_cur_pos_seg*). Set *HT*[*f*(*s_i_*)] to be *new_cur_pos_seg* in *HT*.For each triple (*b_pos*, *e_pos*, *cur_pos*) in *List*, do {If *PV*[*cur_pos*+1].*s* equals to *s_i_*, do {If (*cur_pos*+1) equals to *e_pos*, repeat loops are found. Let *µ* be *b_pos*, *λ* be *e_pos*-*b_pos*+1. Call *n_loops*←Test_RepeatLoops(*SP*, *µ*, *λ*, 2). Return the triple (µ,λ, *n_loops*) and exit this sub-function. Otherwise, let *cur_pos*++ and update this triple in *List*. }Else delete this triple from *List*. }If *cur_pos_seg* equals to *new_cur_pos_seg*-1, repeat loops are found. Let µ be *cur_pos_seg* and λ be 1. Call *n_loops*←Test_RepeatLoops(*SP*, µ, λ, 2). Return the triple (µ,λ, *n_loops*) and exit this sub-function.While *cur_pos_seg* isn’t “null”, do {Generate a candidate triple (*cur_pos_seg*, *new_cur_pos_seg*-1, *cur_pos_seg*) and add it to *List*. Let *cur_pos_seg* = *PV*[*cur_pos_seg*].*pos*.}}No repeat loop pattern is found. Return the triple (“null”, “null”, “null”).**Sub-Function.** Test_RepeatLoops(*SP*, µ, λ, *n_loops*)If μ + (*n_loops* + 1) × λ − 1 ≤ *n* and <*s_μ_*, *s_μ_*_+1_, …, *s_μ_*_+λ-1_> = <*s_μ_*_+*n_loops*×*λ*_, *s_μ_*_+_*_n_loops_*_×*λ*+1_, …, *s_μ_*_+__(*n_loops*+1)×*λ*-1_>, *n_loops*++ and then call *n_loops*←Test_RepeatLoops(*SP*, µ, λ, *n_loops*).Return *n_loops*.

We use a running example to explain the running process of sub-function Find_RepeatLoops(SP). Given a shopping path *SP* = <EA, AB, BC, CD, DE, EA, AB, BE, EA, AB, BE, EG, GD, DK>, the function reads path segments in SP one by one, and the process of finding loop repeat pattern is shown below.

For the first path segment EA, EA cannot be found as a key in empty *HT*, so the key-value pair (EA, “null”) is inserted to *HT*, and the pair (EA, “null”) is pushed onto an empty *PV*. The position of this pair in *PV* is 0. Therefore, the value associated with EA is changed to 0 in *HT*. *cur_pos_seg* (which is “null”) doesn’t equal to *new_cur_pos_seg*-1 (which is -1). *List* still remains empty.

For the second path segment AB, similar operations are done. After operations, the key-value pair (AB, 1) is added in *HT*, and the pair (AB, “null”) is pushed onto *PV*. 

Similarly, after reading the third path segment BC, the fourth CD, and the fifth DE, pairs (BC, 2), (CD, 3), (DE, 4) are inserted into *HT*, and pairs (BC, “null”), (CD, “null”), (DE, “null”) are pushed onto *PV* sequentially. Because *cur_pos_seg* keeps “null”, no candidate is generated.

For the sixth path segment EA, it is found as a key in *HT* and *cur_pos_seg* is 0, which is the position of previous EA in *PV*. Push the pair (EA, 0) onto *PV* and set the value associated with EA (*i.e.*, *HT*[*f*(EA)]) to be 5 in *HT*. Because *cur_pos_seg* is not “null”, a candidate (0, 4, 0) is generated and added to *List*. And then, *cur_pos_seg* = *PV*[0].*pos =* “null”, so no candidate is generated here.

For the seventh path segment AB, the pair (AB, 1) is pushed onto *PV* and *HT*[*f*(AB)] is set as 6. Since there is a loop candidate (*i.e.*, triple (0, 4, 0)) in *List*, we need to compare AB with the next path segment of this candidate (*i.e.*, *PV*[0+1].*s*). Both of them are AB and they are matching, so we set this triple to be (0, 4, 1). Because *cur_pos_seg* is 1, a new candidate (1, 5, 1) is produced and there are two candidates in *List* now.

When reading the eighth one BE, the pair (BE, 7) is pushed onto *PV* and *HT*[*f*(BE)] is 7. For the candidate (0, 4, 1), the next path segment is *PV*[1+1].*s*=BC, which does not match BE. So this candidate should be deleted from *List*. For the candidate (1, 5, 1), the next path segment, *i.e.*, *PV*[1+1].*s*=BC, does not match BE, so this candidate also needs to be pruned. No new candidate is generated, since *cur_pos_seg* equals to “null”.

For the ninth one EA, the pair (EA, 5) is added at the end of *PV* and the value associated with EA is set as 8 in *HT*. Similarly, two new candidates (5, 7, 5) and (0, 7, 0) are obtained and added to *List*.

Similarly, when reading the tenth path segment AB, the pair (AB, 6) is pushed onto *PV* and *HT*[*f*(AB)] is set as 9. For candidates, triple (5, 7, 5) becomes (5, 7, 6), and triple (0, 7, 0) is converted to (0, 7, 1). And two new candidates, *i.e.*, triple (6, 8, 6) and triple (1, 8, 1), are generated.

When reading the eleventh one BE, the pair (BE, 7) is added at the end of *PV* and *HT*[*f*(BE)] is set as 10. For the candidate (5, 7, 6), the next path segment *PV*[6+1].*s* equals to BE, and is the last one in this candidate. Thus repeat loops are found, and µ, λ are 5, 3 respectively. And then, we call Test_RepeatLoops(*SP*, 5, 3, 2). Since <*s*_5_, *s*_6_, *s*_7_> (*i.e.*, <EA, AB, BE>) are not equal to <*s*_11_, *s*_12_, *s*_13_> (*i.e.*, <EG, GD, DK>), *n_loops* which equals 2 is returned. Thus, we have *n_loops* equals 2. 

### 5.2. Function PCP_Filtering (STP)

Given a shopping transaction path *STP* = <(*s*_0_, *t*_0_, *T*_0_), (*s*_1_, *t*_1_, *T*_1_),…, (*s_n_*, *t_n_*, *T_n_*)>, the procedure of filtering palindrome-contained patterns (*i.e.*, *STP*_1_→*STP*_2_→*STP*_3_, where Trans(*STP*_1_), the reverse-order path of Trans(*STP*_2_), and Trans(*STP*_3_) are equal (say *SP*)) is an iterative process of finding the start position of palindrome-contained pattern (say µ), and the number of path segments of *SP* (say *λ*), such that *s_µ_*_+*i*_ = *s_µ_*_+2*λ*-*i*-1,*reverse*_ = *s_µ_*_+2*λ*+*i*_ (*i* = 0,1,…, *λ* − 1; µ + 2*λ* + *i* ≤ *n*), where *s_µ_*_+2*λ*-*i*-1,*reverse*_ is the reverse-order path segment of *s_µ_*_+2*λ*-*i*-1_. If *µ* and *λ* satisfying the above conditions are found, the connections of three fragments <(*s_µ_*, *t_µ_*, *T_µ_*),…, (*s*_µ+λ-1_, *t*_µ+λ-1_, *T*_µ+λ-1_)>, <(*s*_µ+λ_, *t*_µ+λ_, *T*_µ+λ_),…, (*s_µ_*_+2*λ*-1_, *t*_µ+2λ-1_, *T*_µ+2λ-1_)> and <(*s*_µ+2λ_, *t*_µ+2λ_, *T*_µ+2λ_),…, (*s*_µ+3λ-1_, *t*_µ+3λ-1_, *T*_µ+3***λ***-1_)> in *STP* form a palindrome-contained pattern. This procedure is presented in the sub-function Find_PCP(*STP*), and three data structures (*i.e.*, vector of path segment, list of candidate, and list of candidate suffix) are adopted.

*Definition 15.* A *vector of path segment* (say *V*) stores path segments. Suppose a potential palindrome-contained pattern is *SP*→*SP_reverse_*→*SP*. A *list of candidate* (say *LC*) is a list of triple (*b_pos*, *e_pos*, *cur_pos*), and each triple represents a candidate *SP* (*i.e.*, the fragment <*V*[*b_pos*], *V*[*b_pos*+1], …, *V*[*e_pos*]>), where *cur_pos* is the current matching position between *b_pos* and *e_pos*. A *list of candidate suffix* (say *LCS*) is a list of pair (*inter_posi*, *e_posi*), and each pair represents a candidate suffix of *SP* (*i.e.*, the fragment <*V*[*inter_posi*], *V*[*inter_posi*+1], …, *V*[*e_posi*]> ).

Based on the above definition, we have the following Function PCP_Filtering (*STP*):

**Function.** PCP_Filtering(*STP*)**Method:**While palindrome-contained pattern is found, do{(µ, λ)←Find_PCP(trans(*STP*))If palindrome-contained pattern is found, do {For *STP*, combine fragments <(*s*_µ_, *t*_µ_, *T*_µ_),…, (*s*_µ+λ-1_, *t*_µ+λ-1_, *T*_µ+λ-1_)>, <(*s*_µ+λ_, *t*_µ+λ_, *T*_µ+λ_),…, (*s*_µ+2λ-1_, *t*_µ+2λ-1_, *T*_µ+2λ-1_)> and <(*s*_µ+2λ_, *t*_µ+2λ_, *T*_µ+2λ_),…, (*s*_µ+3λ-1_, *t*_µ+3λ-1_, *T*_µ+3λ-1_)> to form a new *STP* according to Definition 13.} }Return *STP***Sub-Function.** Find_PCP(*SP*)Initialize *V*, *LC* and *LCS* as emptySuppose *SP* = <*s*_0_, *s*_1_, …, *s_n_*>. For each path segment *s_i_* in *SP*, do {If *V* is not empty, do {Get the position of the last element of *V* (say *cur_pos_seg*), and compare the last element of *V* with *s_i_*. If they are reverse-order, let variable *reverse-order* be true. Otherwise, let variable *reverse-order* be false. }Else let *reverse-order* be false.Push *s_i_* onto *V*.For each candidate triple (*b_pos*, *e_pos*, *cur_pos*) in *LC*, do {If *cur_pos* is “null”, let *cur_pos* be *b_pos*; otherwise, let *cur_pos* be added by 1.Compare *V*[*cur_pos*] with *s_i_*. If they are same, do {Let this triple be (*b_pos*, *e_pos*, *cur_pos*) and update it in *LC*. If *cur_pos* equals to *e_pos*, then a palindrome-contained pattern is found. Let µbe *b_pos*, λbe *e_pos*-*b_pos*+1, return the pair of µand λ, and exit this sub-function. }Else delete this candidate triple from *LC*. }For each pair (*inter_posi*, *e_posi*) in *LCS*, do {If *inter_posi*-1 0, and *V*[*inter_posi*-1] and *s_i_* are reverse-order, set this pair as (*inter_posi*-1, *e_posi*) in *LCS*, and generate a new candidate (*inter_posi*-1, *e_posi*, “null”) in *LC*. Otherwise, delete this pair from *LCS*. }If *reverse-order* is true, do {Produce a candidate (*cur_pos_seg*, *cur_pos_seg*, “null”) and insert it to *LC*.Generate a candidate suffix (*cur_pos_seg*, *cur_pos_seg*) and add it to *LCS*. } }No palindrome-contained pattern is found. Return the pair of “null” and “null”.

In the following, we illustrate a running example to show the running procedure of sub-function Find_PCP(SP). For instance, given a shopping path SP = <AB, BC, CD, DE, EF, FE, ED, DC, CD, DE, EF, FG>, its process of finding palindrome-contained pattern is as described below: 

For the first path segment AB, simply let *reverse-order* be false and push AB onto *V*. When reading the second path segment BC, we compare the last element of *V*, which is AB, with BC. Because they are not reverse-order, *reverse-order* is false. Then, we push BC onto *V*.

Similarly, we push CD, DE, EF onto *V*. And no candidate or candidate suffix is generated.

For the sixth path segment FE, since the last element of *V* (EF) and FE are reverse-order, *reverse-order* is true. We push FE onto *V*. A candidate (4, 4, “null”) and a candidate suffix (4, 4) are generated.

When reading the seventh path segment ED, *reverse-order* is false, and ED is pushed onto *V*. For candidate (4, 4, “null”), we compare *V*[4] (EF) with ED and they do not match, so we delete this candidate from *LC*. For candidate suffix (4, 4), since *V*[3] (DE) is reverse-order path segment of ED, this candidate suffix becomes (3, 4) and a new candidate (3, 4, “null”) is generated. 

When reading the eighth path segment DC, *reverse-order* is also false, and DC is pushed onto *V*. For candidate (3, 4, “null”), since *V*[3] (DE) and DC do not match we also prune this candidate from *LC*. For candidate suffix (3, 4), *V*[2] (CD) and DC are reverse-order, so this candidate suffix turns to (2, 4) and a new candidate (2, 4, “null”) is produced. 

When reading the ninth path segment CD, *reverse-order* is true and CD is pushed onto *V*. For candidate (2, 4, “null”), since *V*[2] (CD) and CD match, this candidate turns to (2, 4, 2). For candidate suffix (2, 4), since *V*[1] (BC) and CD are not reverse-order, we delete this candidate suffix from *LCS*. Because *reverse-order* is true, a candidate (7, 7, “null”) and a candidate suffix (7, 7) are produced.

When reading the tenth one DE, *reverse-order* is false and DE is pushed onto *V*. For candidate (2, 4, 2), since *V*[3] (DE) and DE are matching, it turns to (2, 4, 3). For candidate (7, 7, “null”), it is deleted for mismatch. For candidate suffix (7, 7), since *V*[6] (ED) and DE are reverse-order, this candidate suffix becomes (6, 7) and a new candidate (6, 7, “null”) is added to *LC*.

Then for the eleventh one EF, *reverse-order* is also false and EF is pushed onto *V*. For candidate (2, 4, 3), since *V*[4] (EF) and EF are matching, this candidate becomes (2, 4, 4). Thus, a palindrome-contained pattern is found, and µ, *λ* are 2, 3 respectively.

## 6. Generation of Synthetic Shopping Transaction Paths

**Figure 8:**
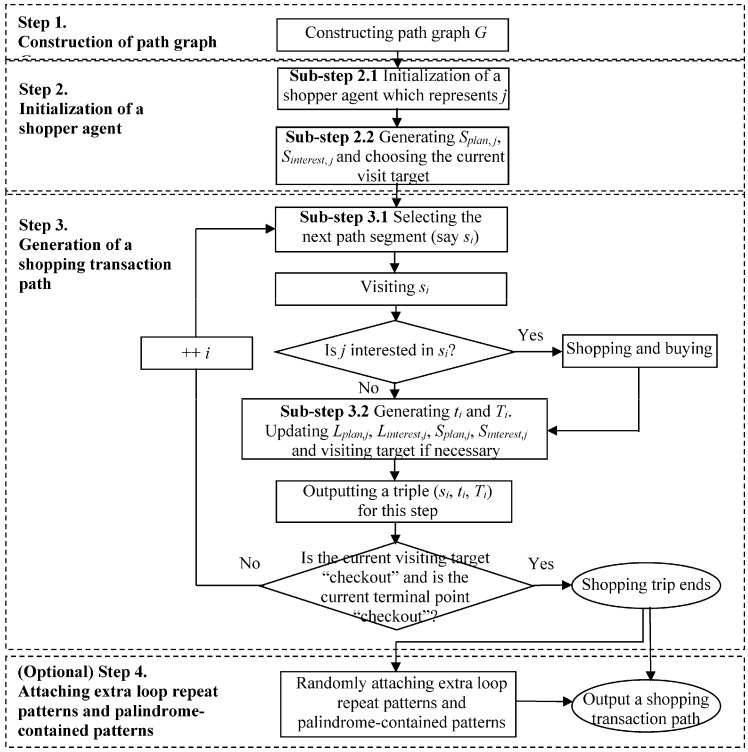
Flow diagram for generating a shopping transaction path.

In order to generate a synthetic workload, we build an agent [41]-based simulator to simulate the scenario of an individual shopping trip. The complete flow diagram for this simulator is shown in Figure 8, which mainly includes four steps: construction of a path graph, initialization of customer agents, generating a shopping transaction path, and attaching extra loop repeat patterns and palindrome-contained patterns. Among them, Step 4 is optional for testing. Steps 2, 3 and 4 can be performed repeatedly for |*D*| times and then a database of shopping transaction paths *D* will be produced. In the following, we discuss these four steps in detail. For the sake of easy reference, the meanings of various variables used in our simulator are summarized in Table 4.

**Table 4:** Meaning of various variables in our simulations.

Notation	Description
*n_terminal_points_, n_path_segments_*	The number of terminal points, path segments in path graph *G* respectively
*n_items_*	The number of different items
*ShoppingTime(i_item_)*	The shopping time for *i_item_*
*j*	A shopper
*speed_normal, j_, speed _j_*	The normal, actual moving speed for *j* respectively
*n_plan, j_*	The number of different planned-purchasing items for *j*
*n_interest, j_*	The number of different items that *j* feels interested in
*L_plan, j_*	A set of planned-purchasing items for *j*
*L_interest, j_*	A set of items that *j* feels interested in
*S_plan, j_*	A set of path segments that *j* plans to visit
*S_interest, j_*	A set of path segments that *j* feels interested in
*μ_speed_normal_*, *σ_speed_normal_*	The mean, the standard deviation of the Gaussian distribution of *speed_normal,j_*
*lower_bound_nplan,j_, upper_bound_nplan,j_*	The lower bound, the upper bound of the uniform distribution of *n_plan, j_* on integers respectively
*n_extra_interest, j_*	The number of additional items (besides items in L_plan, j_) that *j* feels interests in
*lower_bound_nextra_interest, j_, upper_bound_nextra_interest, j_*	The lower bound, the upper bound of the uniform distribution of *n_extra_interest,j_* on integers respectively
*μ_ShoppingTime_*(*i_item,k_*), *σ_ShoppingTime_*(*i_item,k_*)	The mean, the standard deviation of the Gaussian distribution of *ShoppingTime*(*i_item,k_*) respectively
*PerceivedTimePressure_j_*	The perceived time pressure for j
*σ_PerceivedTimePressure_*	The standard deviation of the Gaussian distribution of *PerceivedTimePressure_j_*
*time_s, j_*	Time spent in path segment *s* for *j*
*time_walking, s, j_, time_shopping, s, j_*	Time spent for walking, shopping in path segment *s* for *j* respectively
*Distance(j, s)*	The distance between *j* and path segment s
|*D*|	The number of shopping transaction paths in *D*
*L*	The average number of path segments in shopping transaction paths
*n_LRP_, n_PCP_*	The number of loop repeat patterns, palindrome-contained patterns respectively

### 6.1. Step 1. Construction of Path Graph G

A path graph, the container for customer agents moving in, is constructed in this step. We need to specify the components of a path graph: the set of terminal points of path segments, and the set of path segments. A length table *LT* and a segment-item table *SIT* are also needed to be produced. And then, an item-segment table *IST* can be derived.

### 6.2. Step 2. Initialization of a Shopper Agent

This step includes the following two sub-steps.

#### 6.2.1. Shopper Agent Initialization

In this sub-step, a shopper agent representing an in-store shopper (say *j*) is initialized. Each shopper agent has the following parameters, which need to be specified:

(1) Normal moving speed *speed_normal, j_*

*speed_normal, j_* means the normal moving speed of *j*, which is derived from a Gaussian distribution with mean *μ_speed_normal_* and standard deviation *σ_speed_normal_*.

(2) Number of different planned-purchasing items *n_plan_*_, *j*_

*n_plan_*_, *j*_ represents the number of different items that are planned to be purchased by *j*, and is derived from an uniform distribution on the integers *lower_bound_nplan, j_*, *lower_bound_nplan, j_* + 1, …, *upper_bound_nplan, j_*.

(3) A set of planned-purchasing items *L_plan_*_, *j*_

*L_plan_*_, *j*_ means the set of different items that are planned to be purchased, and can be written as {*i_item_*_,1_, *i_item_*_,2,_ …, *i_item,nplan, j_*}.

(4) Number of different items that *j* feels interested in (say *n_interest_*_, *j*_)

*n_interest_*_, *j*_ is the sum of *n_plan_*_,*j*_ and the number of additional items (besides items in *L_plan_*_, *j*_) that *j* feels interests in (say *n_extra_interest_*_, *j*_). The latter is derived from an uniform distribution on the integers *lower_bound_nextra_interest, j_*, *lower_bound_n__extra_interest, j_* + 1, …, *upper_bound_nextra_interest, j_*.

(5) A set of items that *j* feels interested in (say *L_interest_*_, *j*_)

*L_interest_*_, *j*_ has the form {*i_item_*_,1_, *i_item_*_,2_, …, *i_item,ninterest, j_*}, and each item *i_item_*_,*k*_ is associated with its shopping time *ShoppingTime*(*i_item_*_,*k*_) (*k* = 1, 2,…, *n_interest_*_, *j*_). *ShoppingTime*(*i_item_*_,*k*_) is derived from a Gaussian distribution with mean *μ_ShoppingTi me_*(*i_item,k_*) and standard deviation *σ_ShoppingTi me_*(*i_item,k_*).

(6) Perceived time pressure [42] *PerceivedTimePressure_j_*

*PerceivedTimePressure_j_* represents *j*’s perceived time pressure during a shopping trip, and significantly affects *j*’s moving speed. *PerceivedTimePressure_j_* is also derived from a Gaussian distribution with mean 1 and standard deviation *σ_PerceivedTimePressure_*.

For *j*, his/her actual moving speed *speed_j_* can be simply computed as below:

*speed_j_* = *PerceivedTimePressure_j_* × *speed_normal, j_*. (10)

#### 6.2.2. Generating *S_plan, j_* and *S_interest, j_*, and Choosing the Current Visit Target

In order to decide which direction a shopper agent would like to go, we need to know which path segments *j* plans to visit. These path segments are visit targets for *j*, and *j* will visit these path segments one by one. Here we use *S_plan_*_, *j*_ to represent the set of path segments that *j* plans to visit, and use *S_interest, j_* to represent the set of path segments that *j* feels interested in. According to the item-segment table *IST*, we can derive *S_plan_*_, *j*_ by mapping each item in *L_plan_*_,*j*_ to path segments where this item is sold. Similarly, *S_interest, j_* also can be derived according to *IST* and *L_interest_*_,*j*_.

*Definition 16.* Given a path graph *G*, suppose a shopper *j* is at terminal point *v*, and the start terminal point and the end terminal point of path segment *s* are *s*.*b* and *s*.*e* respectively. Then the distance between the shopper *j* and the segment *s* is defined as below:

*Distance*(*j*, *s*) = min(*shortest_path_length*(*j*, *s.b*), *shortest_path_length*(*j*, *s.e*)) (11)

where function *shortest_path_length*(·, •) means length of the shortest path between two terminal points in *G*, and function min(·, •) represents the minimal one of two values. In the above definition, length of the shortest path between two terminal points in *G* can be obtained using well-known Dijkstra’s algorithm [43,44]. Thus, based on the definition of distance between a shopper and a path segment, we simply use the following method to decide the current visit target.

*Method 1 (deciding the visiting target).* For a shopper *j*, among the elements of *S_plan, j_*, the nearest path segment is regarded as the current visit target. If *S_plan, j_* is empty, “checkout” becomes the moving target. The current visit target remains unchanged until the current visit target has been visited and *S_plan, j_* is updated.

### 6.3. Step 3. Generation of a Shopping Transaction Path

The production of a shopping transaction path can be regarded as a repetitive process of deciding which path segment *s_i_* (*i* = 1,2,…,*n*) should be chosen as the next step, and generating unit time per unit length spent in *s_i_* (say *t_i_*) and the itemset purchased in *s_i_* (say *T_i_*).

#### 6.3.1. Decision on the Next Path Segment

For simplicity, we suppose the walking process of a shopper *j* is as follows: first, *j* selects a visit target, and then he/she walks along the shortest path to the visit target. When he/she reaches the current visit target, he/she needs to decide the next visit target. The process is repeated, until he/she finishes his/her shopping and arrives at “checkout”. Thus, we have the following method for deciding the next path segment.

*Method 2 (deciding the next path segment)*. Given a path graph *G*, if a shopper *j* hasn’t reached the current visit target, the next section along the shortest path to the current visit target is selected as the next path segment for *j*. If *j* arrives at the current visit target, he/she considers and decides the next visit target. Then, the next path segment along the shortest path to the next visit target is chosen as the next section.

In this method, the shortest path to a visit target can be obtained by popular Dijkstra’s algorithm [43,44].

#### 6.3.2. Sub-Step 3.2 Generating *t_i_* and *T_i_*, and Updating *L_plan, j_*, *L_interest, j_*, *S_plan, j_*, *S_interest, j_* and Visiting Target

(1) Generating *t_i_* and *T_i_*

For a shopper *j*, *t_i_* is the quotient of time spent in *s_i_* (say *time_s_i_, j_*) divided by the length of *s_i_* (say *s_i_*.*l*). *time_si_*_, *j*_ consists two parts: time spent for walking in *s_i_* (say *time_walking, s_i_,j_*) and time spent for shopping in *s_i_* (say *time_shopping, s_i_, j_*). *time_walking, s_i_, j_* can be computed as below:

*time_walking, s_i_, j_* = *s_i_.l*/*speed_j_* = *s_i_.l*/(*PerceivedTimePressure_j_* × *speed_normal, j_*) (12)

where *speed_j_* is *j*’s actual moving speed.

For simplicity, the value of *time_shopping, s_i_, j_* depends on whether *j* feels interested in *s_i_* (that is *s_i_ ∈ S_interest_*_, *j*_) or not, and is computed as below:

(13) $${\text{time}}_{\text{shopping},\;s,\;j}=\{\begin{array}{c} \sum_{\forall i_{\text{item}},i_{\text{item}}\in L_{\text{interest},j}˄i_{\text{item}}\in \Gamma_{S_{i}}}\text{ShoppingTime}(i_{\text{item}})\;\text{if}\;S_{i}\;\in \;S_{\text{interest},j}\\ 0\;\;\;\;\;\;\;\;\;\;\;\;\;\text{if}\;S_{i}\notin \;S_{\text{interest},j} \end{array}$$

where *Γ_s_i__* is the itemset sold in *s_i_* and can be obtained from *SIT*.

For *s_i_*, *T_i_* simply equals to the set of items that belong to both *L_plan, j_* and *Γ_s_i__*.

(2) Updating *L_plan, j_*, *L_interest, j_*, *S_plan, j_*, *S_interest, j_* and visiting target

If *s_i_* ∉ *S_interest, j_*, nothing needs to be updated. Otherwise, since *s_i_* has been visited, it should be deleted from *S_interest, j_*. For the reason that *T_i_* has been purchased at *s_i_*, items in *T_i_* need to be removed from *L_plan,j_* and *L_interest_*. If *s_i_* ∉ *S_plan, j_*, *s_i_* also should be pruned from *S_plan, j_*, and the visiting target should be updated further using Method 1, which is given in Section 6.2.2.

### 6.4. Step 4. Attaching Extra Loop Repeat Patterns and Palindrome-Contained Patterns

Producing extra loop repeat patterns and palindrome-contained patterns are exactly the reverse processes of simplifying these two patterns which are presented in Definitions 12 and 13. The methods for producing a loop repeat pattern and a palindrome-contained pattern are described below:

*Method 3 (producing a loop repeat pattern)*. A shopping transaction path, *i.e.*, *STP_prefix_*→*STP_combine_*→*STP_suffix_*, can be transformed to any *STP_prefix_*→*STP*_1_→*STP*_2_→…→ *STP_n_*→*STP_suffix_*, where *STP_combine_=*<(*s*_1_, *t*_1_, *T*_1_), (*s*_2_, *t*_2_, *T*_2_),…, (*s_λ_*, *t_λ_*, *T_λ_*)>, *STP_i_* = <(*s*_1_, *t*_1,*i*_, *T*_1,*i*_), (*s*_2_, *t*_2,*i*_, *T*_2,*i*_),…, (*s_λ_*, *t_λ_*_,*i*_, *T_λ_*_,*i*_)>, *STP_combine_* and all *STP_i_* (*i* = 1,…,*n*) share the same navigation pattern (say Trans(*TSP*) = <*s*_1_, *s*_2_, …, *s_λ_*>), only if the following equations are satisfied:

(14) $$t_{j}=t_{\mathrm{walking}}+\sum_{i=1}^{n}t_{\mathrm{purchasing}}(i,j)\;(j=1,\ldots ,\lambda )$$

*t*_purchasing_(*j*,*i*) = *t_j,i_* − *t*_walking_ (*i* = 1,…,*n*, *j* = 1,…,λ) (15)

(16) $$T_{j}=\overset{m}{\underset{i=1}{\cup }}T_{j,i}\;(j=1,\ldots ,\lambda )$$

where *t*_walking_ is the smallest value of time spent per unit length in this shopping transaction path.

*Method 4 (producing a palindrome-contained pattern)*. A shopping transaction path, *i.e.*, *STP_prefix_*→*STP_combine_*→*STP_suffix_*, can be transformed to any *STP_prefix_*→*STP_1_*→*STP_2_*→*STP_3_*→*STP_suffix_*, where *STP_combine_* = <(*s*_1_, *t*_1_, *T*_1_), (*s*_2_,* t*_2_, *T*_2_),…, (*s_λ_*, *t_λ_*, *T_λ_*)>, *STP_i_* = <(*s*_1_,* t*_1,*i*_, *T*_1,*i*_), (*s*_2_, *t*_2,*i*_, *T*_2,*i*_),…, (*s_λ_*, *t_λ,i_*, *T_λ,i_*)> (*i* = 1, 3), *STP_2_* = <(*s_λ,reverse_*, *t_λ_*_,2_, *T_λ_*_,2_), (*s_λ_*_-1,*reverse*_, *t_λ_*_-1,2_, *T_λ_*_-1,2_), …, (*s*_1*,reverse*_, *t*_1,2_, *T*_1,2_)>, only if the following equations are satisfied:

(17) $$t_{j}=t_{\mathrm{walking}}+\sum_{i=1}^{3}t_{\mathrm{purchasing}}\;(j,i)\;(j=1,\ldots ,\lambda )$$

*t*_purchasing_(*j*,*i*) = *t_j,i_* − *t*_walking_ (*i*=1,2,3, *j*=1,…,*λ*) (18)

(19) $$T_{j}=\overset{3}{\underset{i=1}{\cup }}t_{j,i}\;(j=1,\ldots ,\lambda )$$

where *t*_walking_ is the smallest value of time spent per unit length in this shopping transaction path. Therefore, in order to produce a loop repeat pattern or palindrome-contained pattern, firstly, we randomly choose a fragment of shopping transaction path as *STP_combine_*. And then, transform *STP_combine_* according to Method 3 or Method 4. Multiple loop repeat patterns and palindrome-contained patterns can be produced after executing the above process multiple times.

For a database of shopping transaction paths *D*, five parameters are introduced here: the number of loop repeat patterns (say *n_LRP_*), the number of palindrome-contained patterns (say *n_PCP_*), the average number of path segments in *STP_combine_* for loop repeat patterns (say *λ_LRP_*), the average number of path segments in *STP_combine_* for palindrome-contained patterns (say *λ_PCP_*), the average repeat times in loop repeat patterns (say *n_repeat_*).

## 7. Experimental Results

To assess the performance of the algorithm of identifying the mainstream shopping transaction paths and PFNP-forest algorithm, we conducted several experiments on a PC with a 3.00GHz Intel Core™ 2 Duo E8400 CPU (Santa Clara, CA, USA) and 4GB main memory, running Windows 7 Enterprise Edition. All algorithms are implemented using VC++ 2010. In these experiments, we establish a path graph, which has 159 terminal points and 554 path segments, as an example to generate shopping transaction paths. Without specific explanations, Default values of various parameters used in our simulations are summarized in Table 5. Since the kernel parts of identifying mainstreams are Function LRP_Filtering() (which is used for filtering loop repeat patterns) and Function PCP_Filtering() (which is for filtering palindrome-contained patterns), we test the performance of these two functions.

**Table 5:** Default values of various parameters used in our simulations.

Parameter	Value	Parameter	Value	Parameter	Value
*n_terminal_points_*	159	*lower_bound_nplan, j_*	1	*|D|*	1000
*n_path_segments_*	554	*upper_bound_nplan, j_*	20	*n_repeat_*	3
*n_items_*	3000	*lower_bound_nextra_interest, j_*	0	*n_LRP_*	1000
*μ_speed_normal_*	0.6	*upper_bound_nextra_interest, j_*	8	*n_PCP_*	1000
*σ_speed_normal_*	0.1	*μ_ShoppingTime_(i_item,k_)*	1	*λ_LRP_*	3
*σ_PreceivedTimdPressure_*	0.1	*σ_ShoppingTime_(i_item,k_)*	0.5	*λ_PCP_*	3

### 7.1. Variations of n_LRP_ (or n_PCP_)

**Figure 9:**
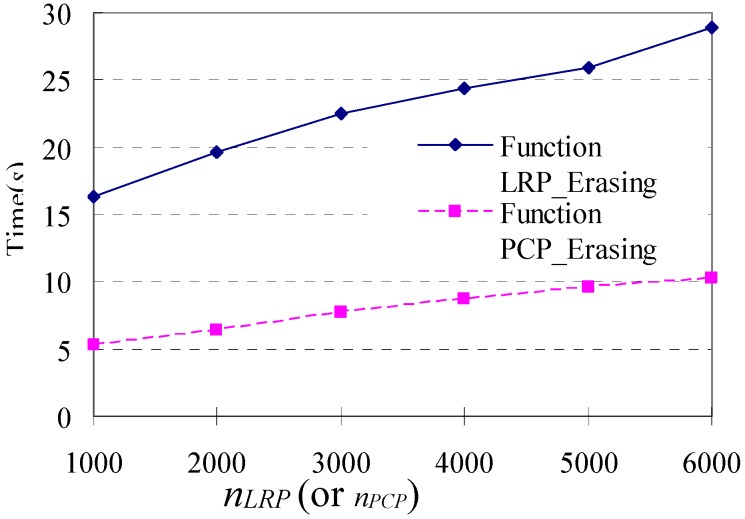
Execution time in response to changes in different *n_LRP_* (or *n_PCP_*).

Firstly, variations of different execution times with different values of *n_LRP_* (or *n_PCP_*) are evaluated and compared. We run the simulation (without Step 4) 1000 times and obtain an original database of shopping transaction paths with *|D|* = 1000 and *L* = 57.4. And then, extra 1000, 2000, 3000, 4000, 5000 and 6000 loop repeat patterns (or palindrome-contained patterns) are attached to the original database with *λ_LRP_* = 3, *λ_PCP_* = 3, and *n_repeat_* = 3, respectively, using Step 4 in simulation. The execution time of Function LRP_Filtering() and Function PCP_Filtering() in response to different *n_LRP_* (or *n_PCP_*) are shown in Figure 9.

We can find that the execution time of Function LRP_Filtering() is about three times that of Function PCP_Filtering() for the same n_LRP_ (or n_PCP_). Both of them increase linearly with the increase of n_LRP_ (or n_PCP_) and have a good scalability.

### 7.2. Variations of *λ_LRP_* (or *λ_PCP_*)

**Figure 10:**
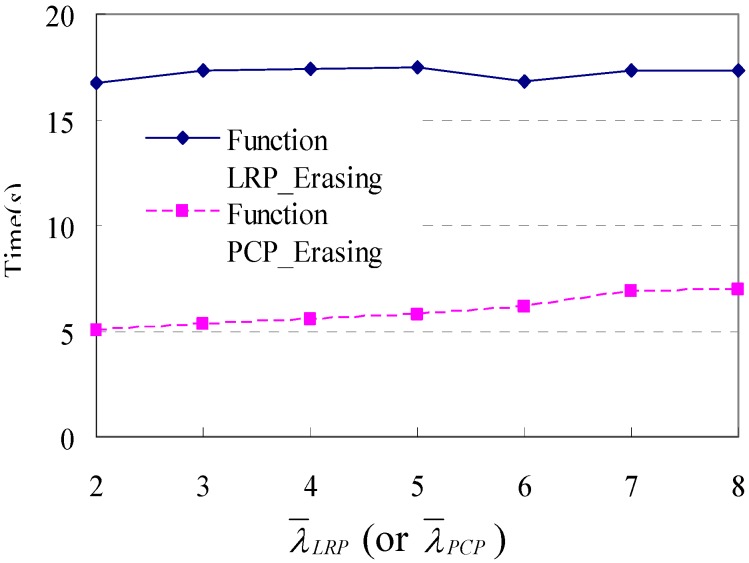
Execution time in response to changes in different *λ_LRP_* (or *λ_PCP_*).

Secondly, we test the scalability of these two functions with the increasing of parameter *λ_LRP_* (or *λ_PCP_*). Similarly, we obtain the original database of shopping transaction paths with *|D|* = 1000 and *L* = 57.4 by running the simulation (without Step 4) 1000 times. Then extra 1000 loop repeat patterns (or palindrome-contained patterns), whose *λ_LRP_* (or *λ_PCP_*) is 2, 3, 4, 5, 6, 7 and 8, are attached to the original database respectively. The impact of *λ_LRP_* and *λ_PCP_* to the execute time is shown in Figure 10. We can find that both these two functions have a relatively stable execution time with the increase of *λ_LRP_* (or *λ_PCP_*).

### 7.3. Variations of |D|

**Figure 11:**
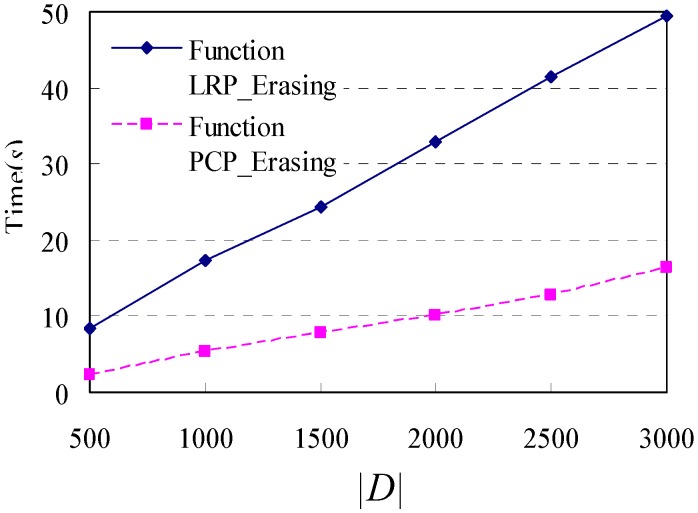
Execution time in response to changes in different *|D|*.

Thirdly, the impact of Variations of *|D|* on the execution time is examined. We run the simulation (without Step 4) 500, 1000, 1500, 2000, 2500, 3000 times, respectively, and obtain the corresponding databases of shopping transaction paths. The values of *L* of these databases are 57.7, 57.4, 57.0, 56.9, 56.9 and 57.0, respectively, which are approximately 57. Then, an extra one loop repeat pattern (or palindrome-contained pattern) with *λ_LRP_* = *λ_PCP_* = 3 and *n_repeat_* = 3 is attached to each shopping transaction path of these databases by using Step 4 in the simulator. The experimental results are shown in Figure 11. It is obvious that the execution time of these two functions increases with the increase of *|D|*, and both of them have a good scalability.

### 7.4. Variations of L

**Figure 12:**
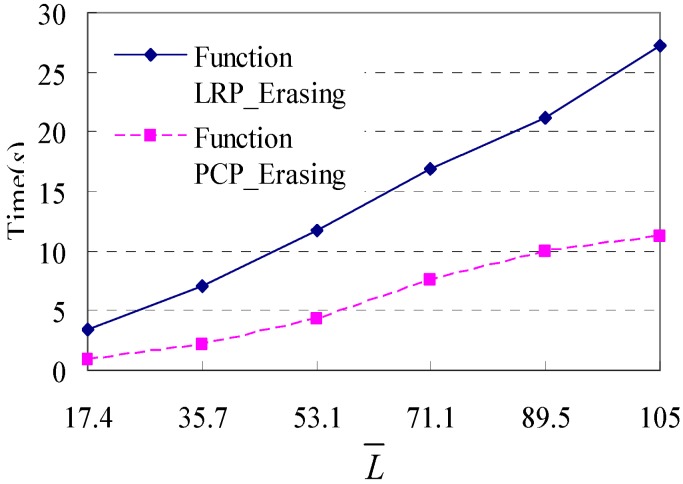
Execution time in response to changes in different *L*.

The fourth test examines the execution performance of these two functions with varying *L*. To obtain different databases of shopping transaction paths with different *L*, we set the value of the pair (*lower_bound_nplan, j_*, *upper_bound_nplan, j_*) to (1, 1), (1, 8), (1, 18), (5, 25), (12, 30) and (20, 36), and running the simulation (without Step 4) 1000 times respectively. Thus we generate six databases of shopping transaction paths whose *L* is 17.4, 35.7, 53.1, 71.1, 89.5 and 105.0, respectively. The difference between successive values of these *L* is approximately 18. Then, we test Function LRP_Filtering() and Function PCP_Filtering() on these databases, and the experimental results are given in Figure 12. We can find that the execution time of these two functions increases in a linear manner, and both of these two functions show a good scalability with the increase of *L*.

### 7.5. Variations of n_repeat_

**Figure 13:**
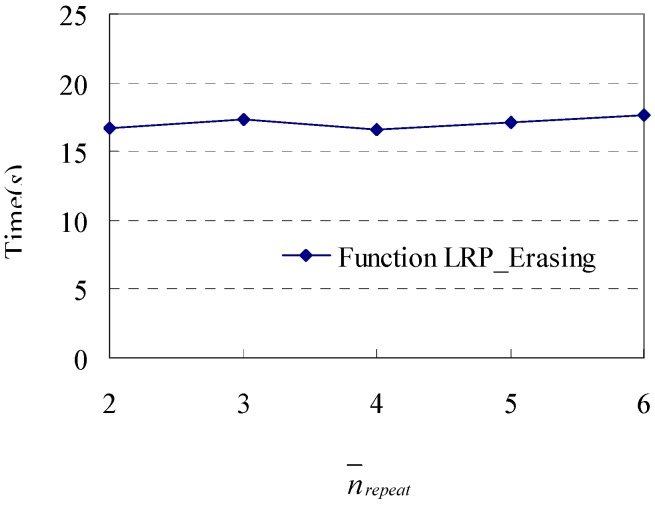
Execution time in response to changes in different *n_repeat_*.

Since only Function LRP_Filtering() has the parameter *n_repeat_*, here we test the impact of variations of *n_repeat_* on the execution time of Function LRP_Filtering(). After obtaining the original database of shopping transaction paths with *|D|* = 1000 and *L* = 57.4, 1000 loop repeat patterns with varying *n_repeat_* (which are 2, 3, 4, 5, 6) are attached to the original database respectively, and then the corresponding databases with varying *n_repeat_* are obtained. We test the varying execution time for these databases and show the experimental results in Figure 13. We find that the execution time is almost stable with different *n_repeat_*. This result also can be obtained by analysing Function LRP_Filtering(). Since varying *n_repeat_* will not change the number of repeat loop patterns that are found, the execution times will not change too much for different *n_repeat_*.

## 8. Contributions toward a Real Supermarket Scenario

The contributions of the framework towards a real supermarket scenario include the following aspects: (1) It provides a feasible way for retail practitioners to record customers’ shopping trajectories associated with their purchasing behaviors using RFID technology; (2) It designs a path graph schema with the support of a segment-item table and item-segment table, which can be used for the mapping between the physical world and the semantic cyber space. After the mapping, the data semantics can be understood by retail practitioners; (3) It offers a practical approach for preprocessing raw in-store RFID data, which contains five steps: data ordering and compression, data merging and anomaly detection, segment extraction, segment reassembling and extracting mainstream shopping transaction paths. Based on this approach, the raw data will become reliable and clean for retail practitioners; (4) It aims at mining actionable navigation patterns from a combination of customers’ shopping paths and their purchasing behavior data. Actionable knowledge is quite useful for decision making [24,25]. For example, we firstly cluster trajectories according to the duration of customers’ stays in the store. Then, practitioners can intuitively explore long “stock-up” trajectories where a long time is spent and many different types of products are purchased by customers. Some interesting patterns may be discovered by data mining algorithms, e.g., shoppers of these “stock-up” trajectories tend to frequently walk through a certain popular spot. Thus, decision-makers may consider offering active services, such as product recommendations and advertising, in that spot.

## 9. Conclusions

In this paper, we use the retail industry as an example to explore the potential of RFID technology for indoor mapping and navigation. In a supermarket scenario, RFID provides the ability to interact with items (*i.e.*, transport carts, trolleys, kegs and valuable products) without physical contact. Thus, item-level RFID infrastructures not only provide item handling efficiency, but also offer a promising way to capture customers’ in-store behavior data and then gain insight into these data using data mining technology. 

In this context, we provide a framework for mining actionable navigation patterns by combining RFID in-door mapping and data mining techniques. In the framework, multi-source in-door RFID data (*i.e.*, shopping path data and RFID-supported customers’ purchasing behavior data) is integrated together for in-depth customers’ behavior analytics. The framework consists of four modules: (1) mapping from the physical space to the cyber space; (2) data preprocessing; (3) data mining mechanism; and (4) knowledge understanding and utilization. Among them, the kernel part, *i.e.*, the scheme of extracting mainstream shopping transaction paths, is discussed in detail. The scheme of identifying mainstreams aims at catching the mainstream path sequences while discarding unnecessary redundant and repeated details, and is quite different from the scheme of extracting maximal forward reference. Two types of redundant patterns, *i.e.*, loop repeat pattern and palindrome-contained pattern, are recognized, and the corresponding algorithms are proposed and evaluated. Experimental results show that the algorithm is efficient and scalable for filtering these redundant patterns. On the whole, this work builds a bridge between indoor positioning and advanced data mining technologies, and provides a feasible way to study customers’ shopping behaviors via multi-source RFID data.
